# Suicide-Related Outcomes Following Gender-Affirming Treatment: A Review

**DOI:** 10.7759/cureus.36425

**Published:** 2023-03-20

**Authors:** Daniel Jackson

**Affiliations:** 1 Psychiatry and Behavioral Sciences, Norton College of Medicine, Upstate Medical University, Syracuse, USA

**Keywords:** suicide, transgender, suicide prevention, transgender youth, transgender health, transgender and gender-diverse, suicide risk

## Abstract

Gender-affirming treatment remains a topic of controversy; of particular concern is whether gender-affirming treatment reduces suicidality. A narrative review was undertaken evaluating suicide-related outcomes following gender-affirming surgery, hormones, and/or puberty blockers. Of the 23 studies that met the inclusion criteria, the majority indicated a reduction in suicidality following gender-affirming treatment; however, the literature to date suffers from a lack of methodological rigor that increases the risk of type I error. There is a need for continued research in suicidality outcomes following gender-affirming treatment that adequately controls for the presence of psychiatric comorbidity and treatment, substance use, and other suicide risk-enhancing and reducing factors. There is also a need for future systematic reviews given the inherent limitations of a narrative review. There may be implications on the informed consent process of gender-affirming treatment given the current lack of methodological robustness of the literature reviewed.

## Introduction and background

Gender-affirming treatment remains a topic of controversy, with many calling for greater access to gender-affirming treatments to foster psychological well-being for transgender, nonbinary, and intersex individuals [1-6]. There is accumulating literature that suggests transgender individuals suffer worse mental health outcomes than their cisgender peers; of particular concern is increased suicidality [4,7-13].

The literature to date reveals concerning trends regarding suicidality in transgender individuals. A high prevalence of suicide attempts and thoughts of suicide occur in transgender youth compared to their cisgender peers [11,12,14]. Transgender US military veterans have more than 20 times higher rates of suicide-related events than cisgender veterans [7]. The prevalence of suicidal ideation and attempts varies by sample [8], with the prevalence of suicidal ideation sometimes as high as 50-75% [4,10,15]. Rates of attempted suicide can reach peaks of 30% and above [4,14,15]. One longitudinal study of over 6,000 transgender individuals in the US indicates that the highest risk of suicide is among those under 18 years of age [9].

Transgender individuals are also at increased susceptibility for various suicide risk-enhancing factors, as a growing body of literature suggests that transgender individuals face a high burden of chronic health conditions [16,17], psychiatric illnesses and their comorbidities [18-20], substance use [21], trauma and victimization [20,22-24], and housing and employment discrimination [25].

In light of this high prevalence of suicidality and the proliferation of gender-affirming treatments, a common argument by advocates of gender-affirming treatments is that such treatments are needed to reduce suicidality [26-29]. This review is the first of its kind to evaluate mental health outcomes from gender-affirming treatments solely from the standpoint of suicidality, with the recognition that this evaluation of suicide-related outcomes pertains to transgender individuals as a single group; however, transgender and gender-diverse individuals comprise a heterogeneous population that may experience varying degrees of health outcomes and biopsychosocial stressors [20].

## Review

Methods

On October 21, 2022, the following search strategy was used in PubMed: ("Suicide"[Mesh] OR suicid*[tiab]) AND ("Sex Reassignment Procedures"[Mesh] OR "sex change*"[tiab] OR "gender change"[tiab] OR "sex reassignment*"[tiab] OR gender reassignment*[tiab] OR "sex confirmation*"[tiab] OR "gender confirmation*"[tiab] OR "gender affirm*"[tiab] OR transitional surgery[tiab] OR "Gonadal Steroid Hormones"[Mesh] OR"Gonadotropin-Releasing Hormone"[Mesh] OR Hormon*[tiab]) AND ("Transgender Persons"[Majr] OR "Gender Dysphoria"[Majr] OR "Gender Identity"[Majr] OR transgender[tiab] OR "gender dysphoria"[tiab] OR "gender identity"[tiab]) AND (following[tiab] OR after[tiab] OR outcome[tiab]).

The search terms resulted in 49 articles, of which the title and abstract were screened for inclusion. Included studies were required to be quantitative, peer-reviewed, published in English, and had an outcome measure of suicidal ideation and/or attempt after gender-affirming surgical procedures (hysterectomy, oophorectomy, mastectomy, phalloplasty, scrotoplasty, and breast, penile, or scrotal prosthesis), hormone treatment (including puberty-blocking treatment), and any combination thereof.

**Figure 1:**
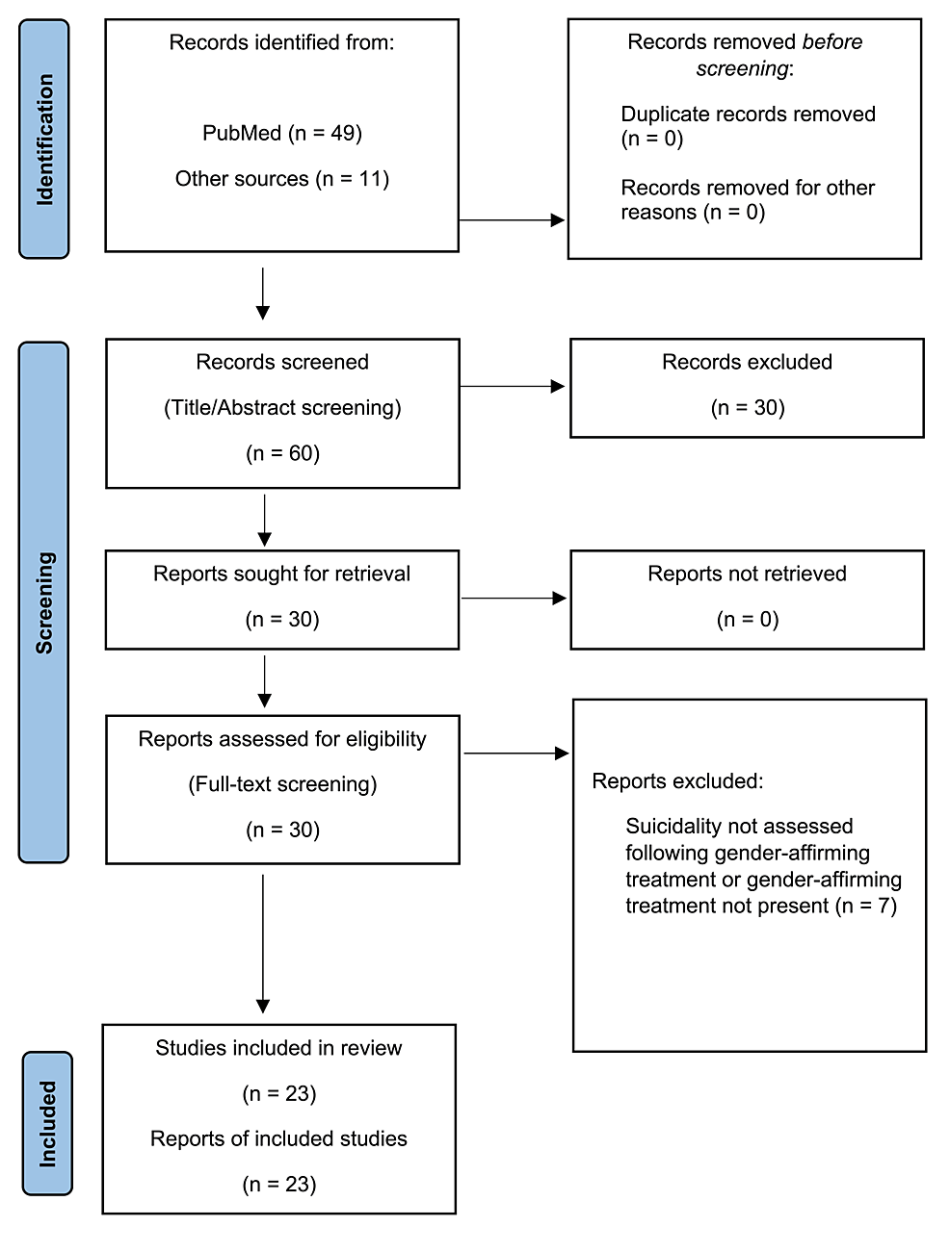
PRISMA flow diagram PRISMA: Preferred Reporting Items for Systematic Reviews and Meta-Analyses. From: Page MJ, McKenzie JE, Bossuyt PM, et al.: The PRISMA 2020 statement: an updated guideline for reporting systematic reviews. BMJ. 2021, 372:n71. doi: 10.1136/bmj.n71 [30].

Out of screening the titles and abstracts of these 49 results for relevance, 19 were evaluated via full-text review for inclusion, of which 15 met the inclusion criteria. Based on references contained in the papers initially reviewed, the full text of an additional 11 papers was screened, with eight meeting the inclusion criteria (Figure 1). The papers that met the inclusion criteria are grouped according to the type of gender-affirming treatment. Most studies that include surgery had patients on cross-sex hormones, but they used surgery as the designation of categorizing outcomes before and after an intervention (Table 1).

**Table 1:** Results MtF: male-to-female; FtM: female-to-male.

	Study type	Treatment type	Gender	Control for time elapsed since treatment	Before and after comparison	Within-groups or between-groups	Measure of statistical significance	Measure of effect size	Correction for multiple testing	Control for psychiatric diagnoses (axis I and II) or the presence of mood disturbance	Control for psychiatric treatment before or after gender-affirming treatment	Control for substance use or abuse	Control for other suicide risk-enhancing or risk-reducing factors	Accounts for death by suicide
Almazan and Keuroghlian (2021) [31]	Cross-sectional survey	Surgery	MtF, FtM, and nonbinary	No	No	Between-groups	Yes	Yes	Yes	Yes	No	No	Age, sex, gender identity, race/ethnicity, employment status, education, sexual orientation, family rejection, income, and health insurance status	No
Bränström and Pachankis (2020) [32]	Total population prospective	Combination or not specified	MtF and FtM	Yes	No	Within-groups	Yes	Yes	No	No	No	No	Legal gender, age, country of birth, education, urbanicity, and household income	No
Chaovanalikit et al. (2022) [33]	Prospective cohort	Surgery	MtF	Yes	Yes	Within-groups	Yes	No	No	No	No	No	No	No
De Cuypere et al. (2006) [34]	Retrospective cohort	Surgery	MtF and FtM	No	Yes	Within-groups	Yes	No	No	No	No	No	No	No
Dhejne et al. (2011) [35]	Population-based matched cohort	Surgery	MtF and FtM	No	No	Between-groups	Yes	Yes	No	No	Yes	Yes	Sex, age, immigration status, and inpatient psychiatric treatment	Yes
Glynn et al. (2016) [36]	Cross-sectional survey	Combination or not specified	MtF	No	No	Both	Yes	Yes	No	No	No	No	Age, ethnicity, and HIV status	No
Heylens et al. (2014) [37]	Prospective cohort	Combination or not specified	MtF and FtM	Yes	Yes	Within-groups	Yes	No	No	No	No	No	No	Yes
Hisle-Gorman et al. (2021) [38]	Retrospective cohort	Hormones (including puberty blockers)	Transgender and gender-diverse	Yes	Yes	Both	Yes	Yes	No	No	Total healthcare contacts per year	No	Sex, total healthcare contacts per year, age at gender-affirming treatment initiation, use of puberty blockers vs. gender-affirming hormones, and parental rank	No
Hughto et al. (2020) [39]	Cross-sectional survey	Combination or not specified	MtF and FtM	No	Yes	Within-groups	Yes	Yes	No	No	No	No	Age, education, and gender-related discrimination	No
Hunt and Hampson (1980) [40]	Cross-sectional survey	Surgery	MtF	No	No	Within-groups	No	No	No	No	No	No	No	No
McNichols et al. (2020) [41]	Cross-sectional survey	Surgery	FtM	No	Yes	Within-groups	Yes	No	No	No	No	No	No	No
Park et al. (2022) [42]	Cross-sectional survey	Surgery	MtF and FtM	No	Yes	Within-groups	No	No	No	No	No	No	No	No
Rehman et al. (1999) [43]	Cross-sectional survey	Surgery	MtF	No	Yes	Within-groups	No	No	No	No	No	No	No	No
Rood et al. (2015) [44]	Cross-sectional survey	Combination or not specified	MtF and FtM	No	No	Between-groups	Yes	Yes	No	No	No	No	Age, race, ethnicity, education, and gender identity	No
Simonsen, Giraldi, et al. (2016) [45]	Retrospective cohort	Surgery	MtF and FtM	No	Yes	Both	Yes	Yes	No	No	No	No	No	Yes
Simonsen, Hald, et al. (2016) [46]	Retrospective cohort	Surgery	MtF and FtM	No	Yes	Both	Yes	Yes	No	No	No	No	No	Yes
Tordoff et al. (2022) [5]	Prospective cohort	Hormones (including puberty blockers)	MtF, FtM, and nonbinary	Yes	No	Between-groups	Yes	Yes	No	Yes	Yes	Yes	Self-reported gender, race, and ethnicity, self-report of conflict with parents due to gender identity or expression, and resilience	No
Tucker et al. (2018) [47]	Cross-sectional survey	Combination or not specified	MtF and FtM	No	No	Between-groups	Yes	Yes	No	Yes	No	No	Age, gender, race, and income	No
Turban et al. (2020) [48]	Cross-sectional survey	Hormones (including puberty blockers)	MtF and FtM	No	No	Between-groups	Yes	Yes	No	No	No	No	Age, gender identity, relationship status, family support, income, sexual orientation, education, and employment	No
Turban et al. (2022) [49]	Cross-sectional survey	Hormones (including puberty blockers)	MtF and FtM	No	Yes	Between-groups	Yes	Yes	Yes	No	No	No	Age, gender, sex, level of family support, sexual orientation, race/ethnicity, income, relationship status, education, employment, and harassment	
van der Miesen et al. (2020) [50]	Cross-sectional survey	Hormones (including puberty blockers)	MtF and FtM	No	No	Between-groups	Yes	Yes	Yes	No	No	No	Age, ethnicity, education, and parent's marital status	No
Wilson et al. (2015) [51]	Cross-sectional survey	Combination or not specified	MtF	No	No	Between-groups	Yes	Yes	No	No	No	No	Age and race/ethnicity	No
Zaliznyak et al. (2021) [6]	Cross-sectional survey	Hormones (including puberty blockers)	MtF and FtM	No	Yes	Within-groups	No	No	No	No	No	No	No	Yes

Results

Combination or Not Specified

Hughto et al. (2020) utilized a cross-sectional, online survey of 288 US-based transgender adults via the Transgender Stress and Health Study. Bivariate and multivariable mixed-effect logistic regression analyses were used.

Participants were asked if they ever had a history of suicide attempt(s) or thoughts of suicide as a dichotomous variable before gender-affirming treatment. Prior to initiating unspecified gender-affirming treatment(s), 73.3% of the sample reported a history of suicidal ideation; this percentage dropped to 43.4% following the initiation of gender-affirming treatment. Prior to treatment initiation, 35.8% of the sample reported a history of suicide attempt(s), and 9.4% reported a history of suicide attempt(s) after initiation of gender-affirming treatment [39].

Adjusted multivariate analyses revealed greater odds of suicidal ideation (adjusted odds ratio (aOR), 3.86; 95% CI, 2.67-5.57; p < 0.001) and suicide attempt(s) (aOR, 5.52; 95% CI, 3.45-8.84; p < 0.001) before gender-affirming treatment compared to after [39]. Odds were adjusted for age, education, and gender-related discrimination. Potential interactions of psychiatric diagnostic history, psychiatric treatment after gender-affirming treatment, substance use, or time elapsed since gender-affirming treatment initiation were not evaluated.

Bränström and Pachankis (2020) conducted a total population study using the Swedish Total Population Register to evaluate the likelihood of mental health treatment following the initiation of hormone treatment or since the last surgical treatment. Hospitalization after a suicide attempt was the measure of suicidality implemented via the International Classification of Diseases, Tenth Revision (ICD-10) codes for intentional self-harm as a primary or secondary diagnosis. The population data from 2015 were utilized to avoid confounding by societal trends over time. As the primary outcome was the likelihood of mental health treatment as a function of time since the initiation of hormone treatment or since the last surgical treatment, the likelihood of mental health treatment that compared before and after gender-affirming treatment was not assessed.

Compared to the general population, transgender individuals had an increased odds of being hospitalized after a suicide attempt (aOR, 6.79; 95% CI, 4.45-10.35); however, a statistically significant relationship was not found for the odds of hospitalization after a suicide attempt after adjusting for the amount of time following the initiation of hormone treatment (aOR, 1.12; 95% CI, 0.97-1.30) or since the last surgical treatment (aOR, 0.87; 95% CI, 0.61-1.24) [32]. The odds ratios were adjusted for legal gender, age, country of birth, education, urbanicity, and household income. The odds ratios were not adjusted for any potential confounding by psychiatric diagnosis, psychiatric treatment besides inpatient hospitalization for a suicide attempt, or substance abuse.

In a subsequently published erratum, the authors noted no statistically significant difference in odds of hospitalization following a suicide attempt between transgender individuals matched by age, legal gender, education, and country of birth who had and who had not received any gender-affirming hormone or surgical treatment. The authors also reported that there was an absence of information that could be gathered on transgender individuals who died by suicide before 2015 [52].

Heylens et al. (2014) compared data from 57 Belgian transgender individuals before and after gender-affirming hormone treatment and surgery. Follow-up data were collected three to six months following the initiation of gender-affirming hormones and one to 12 months following gender-affirming surgery. Data on the history of suicide attempt(s) and thoughts of suicide via a biographic questionnaire were collected for 54 patients before treatment and 42 patients provided data after treatment. The presence of a history of suicide attempt(s) did not reach statistical significance between data collection periods (p-values not provided). One patient died by suicide [37]. There was no accounting for any potential effect of psychiatric diagnostic differences, concurrent psychiatric treatment, substance use, or other suicide risk-reducing or enhancing factors.

Glynn et al. (2016) conducted a secondary analysis of data gathered from a sample of transgender women who engaged in sex work in California. A structured questionnaire was completed by 573 transgender women. Suicidality was measured by “a single dichotomous (yes/no) item (‘Have you ever thought about committing suicide?’).” Over half of the participants (56%) reported a history of ever experiencing suicidal ideation. Bivariate analyses revealed “no significant group differences among… surgery status or hormone use regarding endorsing suicidal ideation or not” [36].

A history of ever experiencing suicidal ideation was associated with “significantly lower levels of psychological and familial social affirmation than those who did not report lifetime suicidal ideation” via independent sample t-tests. Despite the statistically significant results, no correction for multiple testing was done for suicide-related outcomes following gender-affirming treatment (Tukey’s tests were done for pairwise comparisons between racial groups), and effect sizes were not provided; however, they are likely small: receiving “psychological affirmation gender comfort” was associated with 0.5% fewer respondents experiencing suicidal ideation. Receiving “familial social affirmation satisfaction with family support” was associated with 0.11% fewer respondents experiencing suicidal ideation. Of the respondents, 2.89% were more likely to have a history of ever having suicidal ideation if they were of older age. Chi-square analysis demonstrated that white transgender women were more likely to have ever experienced suicidal ideation than other racial/ethnic groups.

Multivariate analyses demonstrated no statistically significant relationship between gender-affirmation treatments and a lifetime history of ever having suicidal ideation. Adjusted odds ratios showed a weak effect size with older age increasing the odds of ever having suicidal ideation. Adjusted odds ratios showed lower odds of ever having suicidal ideation among Latinas and Asian/Pacific Islanders, with Asian/Pacific Islanders having a larger effect size. There was no accounting of any potential confounding relationship of the results with psychiatric diagnostic history, concurrent treatment, substance use, or other suicide risk-reducing or enhancing factors besides age, ethnicity, or HIV status. The reporting of ever experiencing suicidal ideation as a dichotomous variable precluded any analysis of any relationship between the number of suicide attempts or frequency of suicidal ideation before and after any gender-affirming treatment.

Rood et al. (2015) utilized questionnaires from 350 transgender individuals in Virginia to evaluate the potential relationships between discrimination and transition status on suicide risk. Transition status according to the type and extent of treatment was not specified. Suicidality was measured by the question, ‘‘Have you ever thought about killing yourself?’’ as a dichotomous item. Regression analyses were adjusted for demographic variables; psychiatric diagnostic history was not ascertained by the questionnaire and thus was not controlled for [44].

Out of 350 individuals, 64.9% reported a history of ever experiencing suicidal ideation. Adjusted odds ratios revealed higher odds of a history of ever experiencing suicidal ideation in those who planned to pursue transition compared to those with no plan to receive treatment for transitioning (aOR, 2.85; p < 0.01). Those who lived full-time in their gender/had a full social transition had greater odds of ever experiencing thoughts of suicide compared to those with no plan to receive treatment for transitioning (aOR, 2.68; p < 0.01). Individuals who identified as female-to-male (FTM) had greater odds of ever experiencing thoughts of suicide compared to those who identified as male-to-female (MTF) (aOR, 2.48; p < 0.01). Compared to those who never experienced gender-related discrimination and had no plan to receive treatment for transitioning, those who experienced gender-related discrimination and either planned to receive gender-affirming treatment or were already living full-time as their identified gender had an increased odds of ever experiencing thoughts of suicide (aOR, 1.17; p < 0.05).

The authors interpreted these results by heavily relying on Meyer’s minority stress model [53]. When discussing the limitations of the study, there was no mention of a lack of controlling for potential confounding variables of psychiatric diagnostic history, concurrent psychiatric treatment, substance use, or time elapsed since gender-affirming treatment. Furthermore, there was no discussion of the potential limitations on the validity and generalizability of the findings based on the statistical considerations: the adjusted odds ratio for the interaction of discrimination on suicide is of low magnitude (1.17) and vulnerable to the risk of type I error given the lack of controlling for confounding variables. Likewise, the adjusted odds ratios of increased risk of thoughts of suicide for those who lived full-time in their gender (2.68) and those who planned to pursue gender-affirming treatment (2.85) compared to those with no plan to pursue gender-affirming treatment, while of a moderate magnitude, are vulnerable to either type I error or a decreased magnitude given the lack of adequate controlling for confounding variables.

Wilson et al. (2015) conducted a secondary analysis on 314 surveyed transwomen in San Francisco to compare the odds of various health outcomes according to the type of gender-affirming treatment. All but 22 of these individuals had gender-affirming treatment consisting of hormones, genital surgery, breast augmentation, or any combination thereof. Suicidality was measured as a dichotomous variable by asking the respondents if they had ever experienced thoughts of suicide [51].

Compared to those in the sample with no history of gender-affirming treatment, receiving treatment with hormones (OR = 0.2, 95% CI (0.1, 0.5)) or breast augmentation surgery (OR = 0.3, 95% CI (0.1, 0.6)) were associated with lower odds of ever having thoughts of suicide or attempting suicide. Individuals who received genital surgery did not have a statistically significant difference from those who did not receive gender-affirming treatment. The results were adjusted for age and race/ethnicity. There was no correction for any potential relationship with psychiatric diagnostic history, psychiatric treatment, substance use, or time elapsed since gender-affirming treatment, increasing the likelihood that the statistically significant results were vulnerable to a high risk of type I error.

Tucker et al. (2018) conducted a cross-sectional survey of 206 transgender veterans to compare outcomes among those who received a combination of gender-affirming hormones and surgery on both chest and genitals, hormone treatment only, hormone treatment and surgery on either chest or genitals but not both, and those with a history of no gender-affirming treatment. Participants were asked to rate the frequency of suicidal ideation from one (never) to five (very often or five or more times) within the past year. Respondents were also given question nine of the Patient Health Questionnaire-9 (PHQ-9) to assess suicidal thoughts over the previous two weeks at the time of the survey [47].

Mean scores were adjusted for age, gender, race, ethnicity, and annual household income. Analysis of covariance revealed statistically significant results with large effect sizes in lower past-year suicidal ideation for those receiving both genital and chest surgeries vs. those either receiving one surgery type only or gender-affirming hormones only (η2 = 0.051). This pattern of results continued when analyzing suicidal ideation within the past two weeks, with the addition of there being lower scores of suicidal ideation that were statistically significant and with large effect size (η2 = 0.052) for those with both genital and chest surgeries vs. no history of gender-affirming treatment.

An indirect-effects analysis was done to determine if the percentage of variance in suicidal ideation over the past two weeks between groups was due to the amount of depression over the past two weeks while controlling for covariates. An indirect effect was found for those receiving both chest and genitalia gender-affirming surgery vs. those who received no gender-affirming treatment; depression scores predicted 52.3% of the variance in suicidal ideation over the past two weeks. Similar indirect effects were found when comparing receiving surgery in one area alone or receiving gender-affirming hormones alone vs. receiving both chest and genitalia gender-affirming surgery. Psychiatric treatment, substance use, or other risk-reducing or enhancing factors for suicide besides age, gender, race, and income were not considered potential confounders.

Surgery

Chaovanalikit et al. (2022) conducted a prospective cohort study in which 37 transgender women in Thailand were assessed for quality of life and mental health outcomes before and after gender-affirming surgery. Suicidality was measured utilizing the Hamilton Depression Rating Scale (HAM-D). There were statistically significant improvements in quality of life, depression, and self-esteem. There was no correction for multiple testing, measures of effect size, or control for potential confounders such as psychiatric diagnosis, history of psychiatric treatment, substance use, or demographic variables. None of these patients reported suicidal ideation or attempts after treatment [33].

McNichols et al. (2020) conducted a survey of 246 transgender men who underwent any form of masculinizing/gender-affirming surgery at Johns Hopkins. Suicidality was assessed in the survey via the questions, “Do you have a history of any of the following? (check all that apply)” and “If you had any of the following prior to surgery, which of these have improved? (check all that apply)” with “Suicide Attempt” as an answer choice. A history of suicide attempt(s) was reported by 27% of respondents, and 14% of respondents reported an improvement, with p < 0.003. While the survey questions explicitly refer to “Suicide Attempt” as an indication of suicidality, the authors refer to improvements in “suicidal ideation” in the results section [41]. There was no indication of any measurement of the number of suicide attempts before and after masculinization procedures that were more specific than whether they “improved.” There was no accounting for diagnostic history that was clinically determined and verified beyond self-report, current or past psychiatric treatment, substance use, or any interaction of time elapsed since the masculinization procedure as potential confounders. There were no measures of effect size or correction of p-values for multiple testing.

Dhejne et al. (2011) conducted a population-based matched cohort study of 324 Swedish transgender individuals who underwent gender-affirming surgery with controls matched for age, biological sex, and who were residing in Sweden during the time the case person underwent treatment. Immigrant status and history of inpatient psychiatric treatment were more common among transgender individuals than controls, so these were covariates in the calculation of hazard ratios. The two-sided significance value was set at 0.05, with no correction for multiple testing. The adjusted hazard ratio (aHR) of history of suicide attempt(s) among transgender individuals who underwent gender-affirming surgery was 4.9 (95% CI, 2.9-8.5) compared to matched controls across the entire time frame of the cohort (1973-2003). The odds of death by suicide were higher among transgender individuals who underwent gender-affirming surgery (aHR, 19.1; 95% CI, 5.8-62.9). The aHR was 7.9 (95% CI, 4.1-15.3) for the date range of 1973-1988. The aHR did not reach statistical significance for the period of 1989-2003 (aHR, 2.0; 95% CI, 0.7-5.3) [35].

Transgender women were more at risk of suicide attempt(s) than controls of either sex (aHR, 9.3; 95% CI, 4.4-19.9 for female and aHR, 10.4; 95% CI, 4.9-22.1 for male controls). Transgender men were more at risk for suicide attempt(s) compared to male controls (aHR, 6.8; 95% CI, 2.121.6), but the comparison to female controls did not reach statistical significance. The authors state, “[t]his suggests that male-to-females are at higher risk for suicide attempts after sex reassignment, whereas female-to-males maintain a female pattern of suicide attempts after sex reassignment.”

The authors did not correct for multiple testing. While psychiatric morbidity (including substance use) was controlled for in the form of a history of inpatient treatment, different psychiatric diagnostic categories were not taken into account as potential confounders. There was no consideration of any possible interaction of time elapsed since gender-affirming surgery. Most crucially, these findings refer to transgender individuals who received surgery compared to matched controls, not to these transgender individuals before their surgeries or to transgender individuals who have not undergone gender-affirming surgery.

Almazan and Keuroghlian (2021) conducted a secondary analysis of the 2015 US Transgender Survey (USTS). They evaluated 3,559 transgender individuals who underwent gender-affirming surgery of any kind, at least two years prior to responding to the survey. Suicidality was measured as dichotomous variables to whether a participant had thoughts of suicide or had a suicide attempt within the past year. Post-hoc analyses also evaluated the lifetime presence of suicidal ideation and suicide attempt(s). Undergoing gender-affirming surgery was associated with lower odds of suicidal ideation (aOR, 0.56; 95% CI, 0.50-0.64; p < 0.001) and lower odds of suicide attempt(s) (aOR, 0.65; 95% CI, 0.47-0.90; p = 0.009) within the past year compared to those who desired gender-affirming surgery but had not yet received it. The adjusted odds ratio for suicide attempt(s) did not reach statistical significance following the Bonferroni correction, which required a p < 0.002 [31].

Post-hoc analyses revealed that exposure to gender-affirming surgeries and lifetime measures of suicidal ideation or suicide attempt(s) did not reach statistical significance. Patients who received some of their desired gender-affirming surgeries had lower odds of suicidal ideation (aOR, 0.72; 95% CI, 0.63-0.81; p < 0.001) and suicide attempt(s) (aOR, 0.70; 95% CI, 0.53-0.93; p = 0.01) over the past year compared to those who desired gender-affirming surgery but had not received any, with past-year suicide attempts not reaching statistical significant following Bonferroni correction. Patients who received all of their desired surgeries had lower odds of suicidal ideation (aOR, 0.44; 95% CI, 0.38-0.51; p < 0.001) and suicide attempt(s) (aOR, 0.44; 95% CI, 0.28-0.70; p < 0.001) compared to those who desired gender-affirming surgery but had not received any. No interactions of history of mental health treatment besides gender-affirming counseling, substance use history, or time elapsed from surgery were utilized as potential confounders for initial and post-hoc analyses.

Park et al. (2022) conducted a postoperative survey of 15 patients who underwent gender-affirming surgeries from 1975 to 1989 at the University of Virginia. The postoperative data were compared to the preoperative data of 97 patients. Preoperative data revealed that 23.7% of the original sample had a history of suicidal ideation or suicide attempt(s). Of the 15 patients who responded to the postoperative survey, two reported a preoperative history of suicidal attempt(s); of those two, one reported a history of suicidal attempt(s) in the postoperative period. Eight of the 15 respondents reported a preoperative history of suicidal ideation; of these eight, one reported a history of suicidal ideation in the postoperative period [42].

There was no accounting for any possible interaction of psychiatric diagnostic history, psychiatric treatment, substance use, or other suicide risk-reducing or enhancing variables with suicidality. There was no significance testing or measure of effect size. A strength of the study was the gathering of long-term outcome data; however, contacting patients via phone to conduct the survey did not allow the authors to ascertain if any of the clinic’s patients died by suicide following the initial preoperative data collection.

De Cuypere et al. (2006) conducted a long-term follow-up study on 62 Belgians who had received gender-affirming surgery at the Gender Identity Clinic in Gent since 1986. A minimum of one year following surgery was an inclusion criterion for participation in the study. A semi-structured interview assessed suicidality via the rate of suicide attempts. Though not explicitly defined, the rate of suicide attempts was understood for the purposes of this review as the percentage of patients who had ever attempted suicide rather than the frequency of suicide attempts per person. The Diagnostic and Statistical Manual of Mental Disorders, fourth edition (DSM-IV) axis I and II diagnoses were derived from the initial evaluation before surgery; it was unspecified if diagnostic revisions were made at long-term follow-up [34].

The suicide-attempt rate before gender-affirming surgery was 29.3%; following gender-affirming surgery, the suicide-attempt rate decreased to 5.1% (p = 0.004). The authors concluded that MTF patients attempted suicide as a means to cope with stress more frequently than FTM patients based on semi-structured interviews: “The postoperative male-to-females gave the following reasons for their suicide attempts: the end of a relationship (which they perceived as a challenge to their new gender), postoperative complications or an unease with their looks. They are more fragile when they are less credible in their new gender and when they have more pre-morbid psychiatric problems, especially personality disorders.”

Despite the claims made regarding the differences in the contributing factors for suicide attempts between MTF and FTM patients, there were no quantitative data used to support these findings. Despite the extensive gathering of various demographic and clinical data, even including data on social satisfaction before and after surgery and perceived credibility in one’s new gender, these data were not used to evaluate potential effects on differences in suicide outcomes. There was no controlling for any relationship between psychiatric diagnostic history, or the presence of psychiatric treatment on the rate of suicide before and after gender-affirming surgery was undertaken. There was no correction for multiple testing. A potential relationship of the amount of time elapsed since gender-affirming surgery on the rate of suicide was not assessed, though at least a minimum of one year had passed from surgery to the time of the survey.

Simonsen, Giraldi, et al. (2016) and Simonsen, Hald, et al. (2016) analyzed morbidity and mortality of Danish patients before and after gender-affirming surgery from 1978 to 2010. Both studies identified 104 individuals who had undergone gender-affirming surgery according to the Danish National Health Register. According to the Danish Register of Causes of Death, 10 of these 104 individuals had died following gender-affirming surgery from 1978 to 2014. Out of these 10 individuals, two had died by suicide at 19 and 26 years, respectively, following gender-affirming surgery. The studies discussed limitations from a small sample size, including insufficient statistical power [45,46]. Data concerning death by suicide or any other measure of suicidality before gender-affirming surgery were not compiled, preventing any before-and-after treatment comparison.

Rehman et al. (1999) conducted a follow-up study of 28 MTF individuals who had received gender-affirming surgery in New York from 1980 to 1997. Respondents had a minimum of three years post-surgery at the time of data collection. Suicidality was measured via a questionnaire by the item, “Did you have any suicidal thoughts or gestures before or after the surgery?” Two patients reported thoughts of suicide “shortly after surgery.” The authors noted a “marked decrease of suicide attempts” following surgery; however, their questionnaire did not ask about suicide attempts. It may have been that additional interviews were given [43]. Nonetheless, there was no indication that the data were collected through this method, and exact figures were not provided. One patient died by suicide in jail. A comparison via quantitative analysis of suicidality before and after gender-affirming surgery was not provided.

Hunt and Hampson (1980) conducted a follow-up study on 17 MTF individuals who underwent gender-affirming surgery. Two patients attempted suicide following surgery, within a year and six years after treatment, respectively. Both suicide attempts were in “response to the break-up of a relationship” [40]. No comparison of suicidality before and after surgery was undertaken, and the study would likely have been too underpowered to control for possible confounders of suicide risk-enhancing factors.

Hormones

Hisle-Gorman et al. (2021) conducted a retrospective cohort study of 3,754 transgender and gender-diverse (TGD) youth aged eight to 21 years of age in the US military healthcare system. Mental healthcare utilization of TGD individuals was compared before and after the initiation of gender-affirming hormones or puberty blockers. Mental healthcare utilization of TGD individuals was also compared to their cisgender siblings. Suicidality was measured by the presence of a diagnosis of suicidal ideation or self-harm (non-suicidal self-injury or self-harm with suicidal intent not specified). Odds ratios and incidence rate ratios were adjusted for sex, total healthcare contacts per year, age at gender-affirming treatment initiation, use of puberty blockers vs. gender-affirming hormones, and parental rank.

TGD youth had greater odds of receiving a diagnosis for suicidal ideation or self-harm than their siblings (aOR, 7.45; 95% CI, 6.11-9.08). About a quarter of the TGD cohort were on either puberty blockers or gender-affirming hormones. Data were analyzed to compare their mental healthcare utilization from roughly seven years prior to gender-affirming treatment with one-and-a-half years following treatment initiation. The adjusted incidence rate ratio of mental healthcare visits for suicidality was higher following the initiation of gender-affirming care (adjusted incidence rate ratio, 1.74; 95% CI, 1.18-2.56) [38].

The authors noted an increased use of neuroleptics by the transgender cohort, citing concern that the result meant that lack of gender-affirming care may lead to major depressive disorder with psychotic features. The question of whether off-label use of antipsychotics for what was actually comorbid personality pathology, particularly borderline personality disorder, was never addressed, despite that TGD youth had greater odds of a personality disorder diagnosis than their cisgender siblings (aOR, 2.54; 95% CI, 1.71-3.78) and the increasing recognition of personality disorders occurring in adolescence [54,55]. Had the presence of personality disorders been controlled for, it is possible that the higher incidence rate ratio of mental healthcare visits for suicidality following initiation of gender-affirming treatment would not have reached statistical significance.

Tordoff et al. (2022) conducted a prospective, observational cohort study of 104 transgender and nonbinary persons aged 13-20 years at a Seattle gender clinic. Thoughts of self-harm or suicide were assessed via the PHQ-9 question nine; at baseline, 43.3% of patients reported thoughts of self-harm or suicide in the prior two weeks. Potential confounders included as covariates were temporal trends, self-reported gender, race, and ethnicity, ongoing psychiatric treatment, self-report of conflict with parents due to gender identity or expression, any substance use within the past year, and resilience.

Bivariate and multivariate analyses compared mental health outcomes from the 33.7% of participants who did not receive gender-affirming hormone treatment or puberty blockers and the 66.3% of participants who had by the end of 12-month follow-up. Bivariate analyses revealed an association of substance use with increased odds of thoughts of self-harm and suicide (aOR, 2.06; 95% CI, 1.08-3.93). The receipt of puberty blockers or gender-affirming hormones was associated with decreased odds of thoughts of suicide or self-harm (aOR, 0.47; 95% CI, 0.26-0.86). Temporal trends, self-reported gender, race, and ethnicity, ongoing psychiatric treatment, self-report of conflict with parents due to gender identity or expression, and resilience did not reach statistical significance.

Multivariate analysis demonstrated further reduced odds of thoughts of self-harm and suicide associated with the receipt of puberty blockers or gender-affirming hormones (aOR, 0.27; 95% CI, 0.11-0.65). There was an increased likelihood of thoughts of suicide or self-harm for those who did not receive puberty blockers or gender-affirming hormones at six months (aOR, 2.76; 95% CI, 1.22-6.26) but not at the other measured points in time.

This study provides fairly rigorous methods to control for confounding; in addition to the covariates accounted for in multivariate analyses, the authors employed E-value calculations to control for unmeasured confounding. In their supplementary attachment, they state that “the observed OR of 0.27 could be explained away by an unmeasured confounder that was associated with both the PB/GAH and the moderate to severe depression by a risk ratio of 3.25-fold each, above and beyond the measured confounders, but weaker confounding could not do so” [5]. The large effect size observed in this study warrants further investigation, particularly to determine how robust the effect would be after controlling for axis II diagnoses.

Zaliznyak et al. (2021) reviewed the age of first experiencing persistent gender dysphoria, age of social transition, and age of receiving gender-affirming hormone treatment in a sample of 155 transgender women and 55 transgender men in a Los Angeles clinic. All of these patients had socially transitioned and had received gender-affirming hormone treatment for at least a year. Their mental health histories were also taken. Out of the 55 transgender men, 21% had a history of at least one suicide attempt. The authors reported that out of those patients with a history of suicide attempt(s), 10% reported suicidal ideation after receiving gender-affirming hormone treatment or socially transitioning.

The authors appear to designate "Reported Current Feelings of Suicide Ideation" as whether suicidal ideation occurred after initiating gender-affirming hormone treatment or socially transitioning, thereby conflating the current reporting of suicidal ideation in a snapshot of time as the history of any suicidal ideation occurring after gender-affirming hormone treatment or socially transitioning. No patients reported suicide attempt(s) following gender-affirming hormone treatment or socially transitioning. There were no results given on the average amount of time following transitioning and suicide measures, nor were there tests of statistical significance.

The results for transgender women were reported similarly. Of 155 transgender women, 30% reported a history of suicide attempt(s); 27% of those who had a history of suicide attempt(s) reported current suicidal ideation (though later described as occurring after initiating gender-affirming hormone treatment or socially transitioning). No patients reported suicide attempt(s) following transitioning. There were no results given on the average amount of time following transitioning and suicide measures, nor were there tests of statistical significance.

The authors did not indicate whether they reviewed clinic records for any patients who died by suicide following gender-affirming hormone treatment or socially transitioning. There was no consideration of the effect of confounding diagnoses on the suicidality measures. Nevertheless, the authors conclude: “Given the high prevalence of suicidality, depression, and anxiety among transgender communities, it follows that proper measures should be taken to address the underlying condition − untreated GD [gender dysphoria]” [6].

Turban et al. (2022) examined data from over 21,000 transgender adults from the 2015 USTS. Suicidality was ascertained by inquiring whether there was any suicidal ideation with or without a plan, suicide attempt(s), or suicide attempt(s) requiring hospitalization over the year prior to the survey being taken. Individuals were asked about various demographic and other confounding variables, but any current or prior mental health treatments besides hospitalization secondary to suicide attempt(s) were not gathered and controlled for.

Those who received gender-affirming treatment during adolescence and adulthood were compared to those who desired access to these treatments but never received them. Access to these treatments in early adolescence was associated with lower odds of suicidal ideation over the past year (aOR, 0.4; 95% CI, 0.2-0.6; p < 0.001) compared to those who desired but did not attain these treatments. For late adolescence (aOR, 0.5; 95% CI, 0.4-0.7; p < 0.0001) and for adulthood (aOR, 0.8; 95% CI, 0.7-0.8; p < 0.0001), there were also lower odds of suicidality over the year preceding the survey for those who had access to gender-affirming hormones during those periods of life [49].

Post-hoc analyses revealed that access to gender-affirming hormones during adolescence rather than adulthood was associated with lower odds of suicidality (aOR, 0.7; 95% CI; 0.6-0.9; p = 0.0007); there was no difference when comparing early vs. late adolescence. As mentioned, any current or prior mental health treatments besides hospitalization secondary to suicide attempt(s) were not gathered and controlled for. The authors tried to assess a potential confounding of mental-health differences within the sample by examining those who had a lifetime history of suicidal ideation but none over the past year. There were greater odds of a lifetime history of suicidal ideation (aOR, 1.4; 95% CI, 1.3-1.5; p < 0.0001) but none in the past year for those who accessed gender-affirming hormones in adulthood. Such a comparison in adolescence did not reach statistical significance.

The authors stated that a post-hoc analysis was done by examining those who had a lifetime history of suicidal attempt(s) but none over the past year; however, the results of such an analysis were not described. It is possible that assessing the confounding of mental-health differences by comparing suicidality over the past year to a lifetime history is insufficient. There will be a higher likelihood of the presence of lifetime suicidal ideation but none for the past year not just due to mental health differences but as a function of increased age, i.e., there is a possibility that those who received gender-affirming hormones 30 years ago have a higher chance of a lifetime history of suicidality compared to those who received such treatments five years ago. Additionally, older individuals may have the benefit of potentially having a longer period of time receiving mental health treatment, which may account for no suicidality over the past year. There was no information from those who died by suicide. Finally, there was no accounting for effects due to psychiatric diagnostic history.

Puberty Blockers

Turban et al. (2020) analyzed data from the 2015 USTS to include “3,494 individuals between the ages of 18 and 36 who ever wanted pubertal suppression as part of their gender-affirming medical care” as an adolescent. The results indicated that 89 (2.5%) of this sample received puberty blockers. Univariate analyses indicated lower odds of lifetime suicidal ideation as well as suicidal ideation within the past year for those who received puberty blockers. Multivariate analyses revealed that the receipt of puberty blockers “was associated with decreased odds of lifetime suicidal ideation” (aOR, 0.3; 95% CI, 0.2-0.6). Suicidal ideation within the past year did not reach statistical significance. Lifetime suicide attempts did not reach statistical significance depending on receipt of blockers in univariate analyses and thus were not assessed with multivariate analysis [48]. The presence of mental health treatment, substance use, or psychiatric diagnostic history was neither mentioned nor controlled for.

Van der Miesen et al. (2020) compared outcomes at a gender clinic in the Netherlands between a sample from the general population: 272 transgender adolescents at referral who had not begun puberty blockers, and 178 adolescents who were currently on puberty blockers. Suicidality was measured by items asking, “I deliberately try to hurt or kill myself” and “I think about killing myself” [50].

The control group and those who were currently on puberty blockers did not have any statistically significant difference in suicidality, whereas those who were referred to the clinic but had not begun puberty blockers scored higher in suicidality than the other groups, but Cohen’s *d* revealed small effect sizes. There was neither mention nor control for psychiatric diagnostic history, substance use, or current psychiatric treatment.

Discussion

The majority of the 23 studies reviewed claimed that various forms of gender-affirming treatment were associated with reductions in suicidality; however, the validity and robustness of their results suffered from either a lack of measures of statistical significance and effect size, correction for multiple testing, controlling for psychiatric diagnostic makeup or psychiatric treatment history, substance use, the interaction of time since receiving gender-affirming treatment, or any combination of these. The two studies that showed an increase in suicidality for those who received gender-affirming treatment suffered from many of the same problems in validity and robustness. Additionally, one of these studies did not compare suicidality outcomes before and after treatment but rather to the general population [35], and the other [38] yielded a small effect size that would likely constitute little clinical relevance; moreover, its results may not have reached statistical significance if there was adequate controlling for confounders.

Controlling for a potential effect of psychiatric diagnoses or degree of mood disturbance was undertaken by three of the studies reviewed [5,31,47]. The need to control for comorbid psychiatric diagnoses or degree of mood disturbance is highlighted by the findings of Tucker et al. (2018). Through indirect analysis, they found that depression scores predicted over half of the variance in suicidality over the past two weeks before their sample responded to the survey. The lack of accounting for psychiatric comorbidity and other dynamic suicide risk-enhancing factors may be the greatest limitation in the body of literature to date regarding suicidality outcomes following gender-affirming treatment.

The presence, type, and timing of psychiatric treatment history represent a potential confounder that was not considered by the majority of studies. Three of the reviewed studies accounted for some form of psychiatric treatment [5,35,38]. Hisle-Gorman et al. (2021) controlled for the total healthcare contacts per year (inpatient and outpatient), Dhejne et al. (2011) controlled for inpatient psychiatric treatment, and Tordoff et al. (2022) controlled for “ongoing mental health therapy.” There is accumulating evidence of the efficacy of psychiatric treatments that may lower the risk of suicide [56-58]. It would be beneficial for future studies to collect data for psychiatric treatment both before and after gender-affirming treatments.

Comorbid substance use has been well-documented as a concern for TGD individuals [19-21,59-61]. In addition to substance use being a dynamic risk factor for suicide [62,63], this relationship is borne out for TGD individuals as well [24]. Only two of the studies reviewed accounted for substance use [5,35], revealing a glaring risk of type I error in the literature, as access to gender-affirming treatment may or may not also serve as a proxy to access to other medical treatments, such as treatment for substance use.

Given that the 23 studies spanned a wide range of locations and dates conducted, it is not surprising that a uniform measure of suicidality was not employed across studies. An evaluation of the number of suicide attempts before and after gender-affirming treatment will likely be the most robust measure for suicidality rather than the presence and frequency of thoughts of suicide, particularly measures of suicidal ideation significantly limited in the expanse of time, such as the PHQ-9 question nine employed in Tordoff et al. (2022) and Tucker et al. (2018). A suicide attempt represents a more circumscribed occurrence, thus more easily and reliably quantifiable than thoughts of suicide; however, given that suicide attempts are a rarer phenomenon, the use of this outcome variable alone would yield less power and increase the risk of type II error. Nonetheless, the presence of thoughts of suicide at distinct points in time may be confounded by a diverse experience of such thoughts by individuals. For instance, individuals may be aware of a nearly ever-present sense of suicidal ideation, particularly in the presence of axis II pathology rather than a significant stressor or exacerbation of axis I pathology [64].

The potential confounding nature of utilizing the presence of suicidal ideation as the sole measure of suicidality may be reflected in the literature reviewed. For example, Almazan and Keuroghlian (2021) reported a lack of a statistically significant relationship between gender-affirming surgery and suicide attempts within the past year or the lifetime number of suicidal ideation. However, while there was not a statistically significant relationship with lifetime suicidal ideation, there was a statistically significant relationship with suicidal ideation within the past year. To have three measures of suicidality not reach statistical significance but suicidal ideation within the past year to reach statistical significance may represent multiple possibilities: suicidal ideation may be a more sensitive measure of suicidality as it is more prevalent and thus has more statistical power. Conversely, the presence of a high risk of type I error associated with recall bias and the potential inherent unreliability of suicidal ideation as a measurable construct may be detractors of its use. Finally, differing results according to suicidal ideation vs. attempts of suicide may represent the underpowered nature of the reporting of suicide attempts, which may represent the presence of a high risk of type II error.

The need for clear, objective reporting of suicide risk in transgender persons, including any change attributed to gender-affirming treatment, is highlighted further by the immense difficulty psychiatry as a field has in accurately predicting suicide risk. Even for at-risk populations, suicide attempts and parasuicidal behaviors are statistically rare enough to make it “impossible to predict on the basis of risk factors either alone or in combination” one’s risk of suicide [65].

A dearth of high-quality studies that evaluate outcomes in suicide following gender-affirming treatment poses severe limitations on the extent of claims made during the informed consent process for gender-affirming treatment. An abundance of claims that are not backed by evidence does not represent quality empirical evidence but rather guidelines endorsed by various medical organizations. Just as in practice guidelines for the assessment and treatment of patients at risk for suicide, “practice guidelines do not represent the standard of care, much less for a fact-specific case in litigation” [66].

Clinical judgment, rather than an indiscriminatory tabulation of risk-enhancing factors for suicide, will ultimately be needed, as “no study has identified one specific risk factor or set of risk factors as specifically predictive of suicide or other suicidal behavior” [65]. Risk-enhancing factors for suicide may act in a synergistic manner, with mood disorders, substance use, physical and sexual abuse, minority sexual orientation, disturbed family relationships, parental psychopathology, and various precipitating stress events [67] leading to near-infinite permutations of suicide risk that is ultimately expressed and unique on an individual level. This is especially the case for TGD individuals, for they constitute “heterogeneous groups of individuals with multiple intersecting identities” [20,59] that may contribute to different levels of risk for suicide.

Such permutations of suicide risk reinforce the need to control for various confounders, which is pervasively lacking in the literature to date. Most studies have ignored complex relationships among various risk factors for suicide, despite literature that suggests a nuanced intersection of these factors with suicide, such as victimization and substance use [24]. Given the heterogeneity of risk factors for this population [20,59], adequate control for confounding variables is needed to represent as accurately as possible the variance that can be attributed to gender-affirming treatment on suicide-related outcomes for transgender individuals as a whole and according to other defining characteristics.

In addition to trauma and abuse, other psychosocial stressors, “such as sudden unemployment, interpersonal loss, social isolation, and dysfunctional relationships, can increase the likelihood of suicide attempts as well as increase the risk of suicide” (“Practice Guideline for the Assessment and Treatment of Patients With Suicidal Behaviors,” 2006). It is notable that Tordoff et al. (2022) reported that conflict with caregivers over gender identity did not have a statistically significant relationship with thoughts of suicide, whereas Glynn et al. (2016) reported a statistically significant increase in suicidal ideation for those with less affirmation by one’s family. Additionally, Almazan and Keuroghlian (2021) reported lifetime suicide attempts and thoughts of suicide were not statistically significant with familial rejection as a covariate, potentially meaning that familial rejection accounted for some of the variances in suicide risk. The variety of findings regarding any potential effect of familial conflict on suicide may represent type I error, the unreliability of thoughts of suicide as a measure compared to suicide attempts, and/or the heterogeneous nature of the TGD population.

The collection of data that includes long-term follow-up is ideally suited to take into account the effects of a transgender individual’s time course, which may include a “honeymoon period” after receiving gender-affirming treatment [34]. Equally important is the controlling of time elapsed before and after gender-affirming treatment with regards to suicidality; otherwise, the number of suicide attempts or frequency of thoughts of suicide may be falsely lowered if the relative time after gender-affirming treatment is less than the pre-treatment period. However, the majority of studies did not control for the amount of time elapsed.

Limitations

The limitations inherent in a narrative review format are noted, particularly the absence of a second, independent reviewer for the inclusion and exclusion of studies as well as the lack of a systematized evaluation of publication bias and methodological rigor. Moreover, a single database was utilized, albeit with fairly extensive search criteria. Future systematic and/or scoping reviews are needed. Finally, this review may have limited generalizability. The studies included in this review span multiple countries, cultures, and decades; furthermore, TGD individuals comprise a heterogeneous group.

## Conclusions

There is a need for continued research on suicidality outcomes following gender-affirming treatment. Future research that incorporates multiple measures of suicidality and adequately controls for the presence of psychiatric comorbidity, substance use, and other suicide risk-enhancing factors is needed to strengthen the validity and increase the robustness of the results. There may be implications for the informed consent process of gender-affirming treatment given the current lack of methodological robustness of the literature reviewed.
